# Challenges in Diagnosis and Management of Mastoid Osteosarcoma: A Case Report and Review of Literature

**DOI:** 10.1155/crot/6035926

**Published:** 2025-12-12

**Authors:** Abdullah Aldaihani, Yousef Bolous, Martin Bullock, Nael Shoman, Jonathan Trites

**Affiliations:** ^1^ Division of Otolaryngology Head and Neck Surgery, Department of Surgery, Dalhousie University, 5850 University Avenue, Halifax, B3K 6R8, Nova Scotia, Canada, dal.ca; ^2^ Faculty of Medicine, Dalhousie University, 5850 University Avenue, Halifax, B3K 6R8, Nova Scotia, Canada, dal.ca; ^3^ Department of Pathology, Dalhousie University, 5850 University Avenue, Halifax, B3K 6R8, Nova Scotia, Canada, dal.ca

**Keywords:** mastoid process, osteosarcoma, otoloaryngology

## Abstract

**Background:**

We report a case of osteosarcoma of the mastoid process.

**Methods:**

The tumor was identified after the patient presented with progressive hearing loss 5 years after radiotherapy treatment for parotid mucoepidermoid carcinoma. A CT scan revealed a mass invading the middle ear and mastoid process. The diagnosis of osteosarcoma was confirmed by biopsy.

**Results:**

The mass was surgically excised, and the patient was treated with postoperative cisplatin and doxorubicin. The patient also developed a postauricular abscess that was treated with incision and drainage and antibiotics. This abscess ultimately resolved, and the patient is otherwise well postoperatively.

**Conclusion:**

This case demonstrates the extent to which osteosarcomas can invade into surrounding tissues as this one had reached the middle ear. Moreover, this case highlights the need for multicenter studies to develop an approach to treatment of these rare tumors as they are not well studied currently.

## 1. Introduction

Osteosarcomas are malignant neoplasms of mesodermal origin that produce osteoid tissue. These are the most common primary malignant tumors of the bone, typically arising from the metaphysis of long bones [1]. Osteosarcomas are relatively rare, accounting for approximately 1% of all head‐and‐neck cancers and represent < 10% of all osteosarcomas [2]. Mastoid osteosarcoma may compose 2% of head and neck osteosarcomas, with the majority of cases occurring in the mandible and maxilla [3]. Typical presentation varies based on tumor location; however, the majority present with a mass. Previous studies report an association with prior radiotherapy, Paget’s disease, and trauma [4]. Diagnosis is based on clinical findings, biopsy, and imaging. Surgery is indicated for symptomatic or cosmetically unacceptable osteomas [5]. Only 4 cases have been previously reported to occur in the mastoid process of the temporal bone [4, 6–8]. The mainstay of treatment is surgery, since this offers the best overall survival rate [3, 9]. Adjuvant postoperative radiotherapy is indicated for cases with close or positive margins. The role of adjuvant chemotherapy remains clarified [10]. Here, we report a rare case of mastoid osteosarcoma in a patient with a prior history of radiotherapy, excision and neck dissection, and chemotherapy for parotid mucoepidermoid carcinoma.

## 2. Case

A 55‐year‐old female lady with a known history of a 2.5‐cm, low grade mucoepidermoid carcinoma of the right parotid gland, with focal positive margins, focal perineural invasion, and microscopic extraparotid extension that involved the surrounding skeletal muscle. In addition, 1/6 lymph nodes and 0/20 lymph nodes were involved in right Level II node dissection. She underwent a parotidectomy in April 2014 and received adjuvant radiotherapy with 6600 cGy over 33 fractions delivered between July and September 2014. Five years later, she presented with progressive hearing loss in the right ear; she was referred to the otology clinic, and on examination, she was found to have a red hue in the middle ear. A subsequent CT scan revealed destructive soft tissue density centered at the right mastoid air cells. The differential diagnosis included osteosarcoma, chondrosarcoma, osteochondroma, giant osteoma, organizing scalp hematoma, and parosteal osteogenic sarcoma. A right mastoid content biopsy was done, and it contained multiple fragments of a high‐grade spindle cell and epithelioid malignancy, throughout which are irregular seams of osteoid and mature bone, as well as tumor giant cells and osteoclast‐like giant cells, as shown in Figure 1(b). Immunohistochemistry results are diffuse strong positivity for vimentin, partial moderate positivity for AE1/AE3 and CK8/18 (similar expression), and focal weak membranous positivity for CD30. The following immunostains are negative: leukocyte common antigen, p40, CK5/6, CD3, CD20, S100, and HMB‐45, as shown in Figure 1(a). Those findings were those of a high‐grade osteosarcoma. Two months later, the lesion was excised without preoperative neoadjuvant therapy. The excised specimen consisted of a fragmented lesion with invasion into the mastoid process, posterior ear, and posterior scalp. The pathology report revealed this specimen to be an osteosarcoma with 0/1 lymph nodes involved and a mitotic range 44 per 10 high powered field.

Figure 1(a) Low power image showing the osteosarcoma infiltrating bone. The tumor has a cellular component with abundant strands of pale pink stroma, which is osteoid, characteristic of an osteosarcoma (H&E, 40x). (b) Photomicrograph showing a high‐grade malignancy with mixed epithelioid and slightly spindle‐shaped tumor cells. Immature, focally calcified bone is noted on the right side of the image, and there are several benign osteoclast‐like giant cells (lower‐mid field). Pale pink osteoid is also noted between the tumor cells (H&E, 200x).

**Figure:**
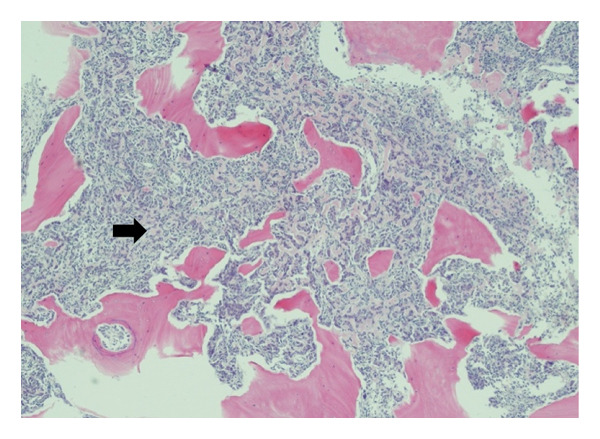
(a)

**Figure:**
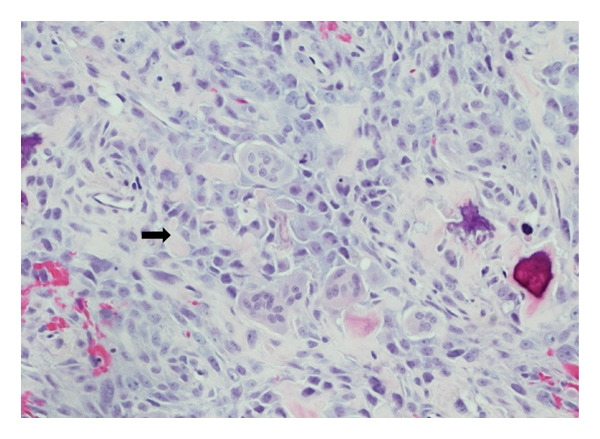
(b)

Postoperatively, the patient received a first cycle of chemotherapy (doxorubicin and cisplatin) 2 months after the surgery. Restaging CT head, chest, abdomen, and pelvis was conducted after completion of the first cycle. There was no evidence of metastatic disease, as shown in Figure 2(a). However, there was interval progression and expansion of a coincidental left temporal osteosarcoma that demonstrated invasion of the posterior and right middle cerebral fossa, as shown in Figure 2(b), and the external and middle ear spaces, as shown in Figure 2(d). The right facial nerve was also involved, as shown in Figure 2(c). She completed 3 cycles of chemotherapy and developed a pancytopenia with every cycle of chemotherapy. She was admitted once for observation and blood transfusion due to severe febrile neutropenia. In addition, she developed a postauricular abscess that was drained on an outpatient basis and started a course of amoxicillin‐clavulanate. Three weeks later, the abscess resolved, and she remains to be followed in the outpatient Otolaryngology clinic every 3 months. However, unfortunately the patient passed away 9 months later due to complications of COVID‐19 virus.

Figure 2CT head: (a) coronal cut, (b) axial cut/bone window, (c) axial cut/soft tissue window, and (d) axial cut with contrast, shows there is a 6.1 cm AP × 3.5 cm TR × 5.3 cm CC, lobulated, heterogeneous mass originating in the region of the right mastoid. There is complete destruction of the right mastoid air cells by the mass. In addition, there is an erosion of the temporal bone and extension into the middle cranial fossa.

**Figure:**
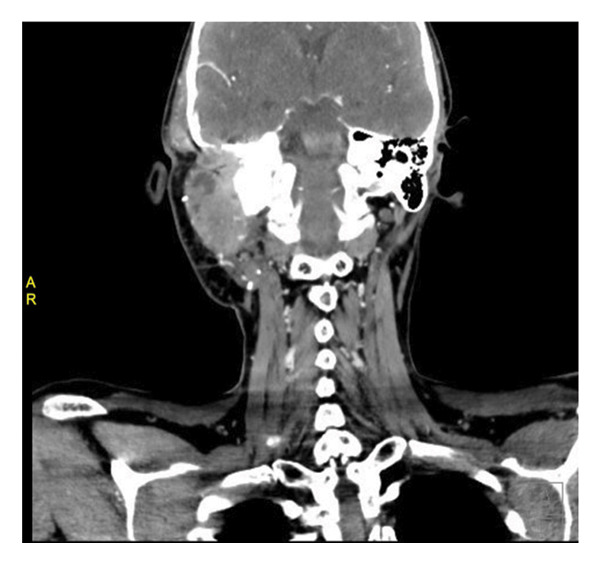
(a)

**Figure:**
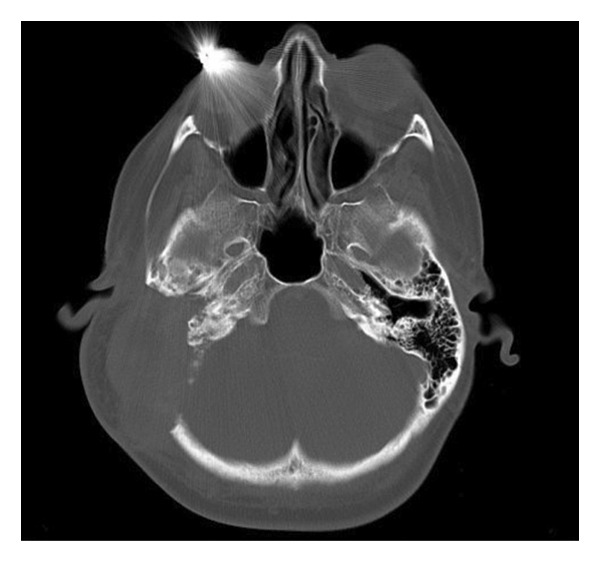
(b)

**Figure:**
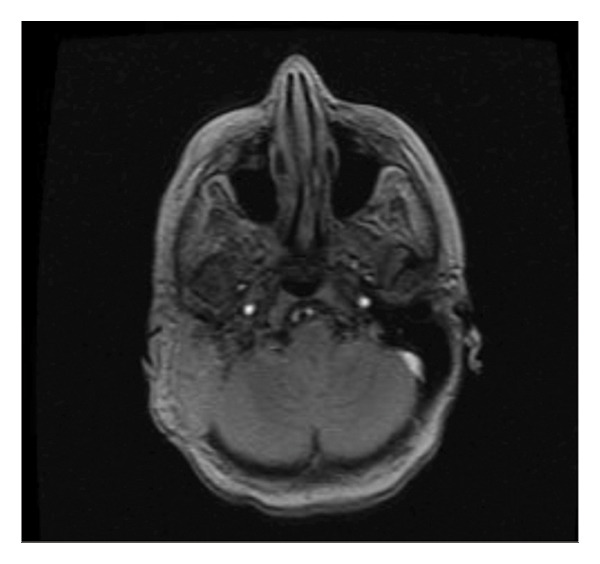
(c)

**Figure:**
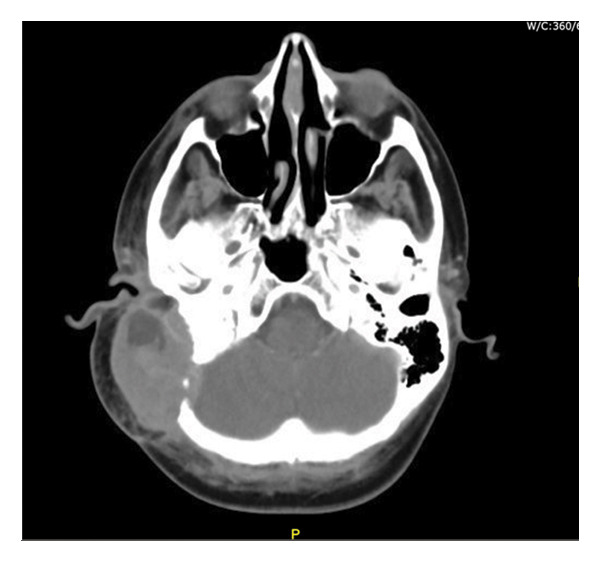
(d)

There is also extension of the mass into the external auditory canal and middle ear. There is obliteration of the scutum, epitympanum, and ossicles. The mass abuts the tympanic segment of the facial nerve with apparent bony erosion.

## 3. Discussion

There are limited reports of osteosarcoma involving the middle ear and mastoid process. In addition, this case presented with a previous history of right parotid mucoepidermoid carcinoma treated with radiotherapy. Brusić et al. reported a similar case where a 75‐year‐old female with a history of radiation therapy for mucoepidermoid carcinoma of the parotid gland after total parotidectomy and radical neck dissection [6]. Hsieh et al. reported a case of paraosteal osteosarcoma of the mastoid process following radiotherapy for nasopharyngeal carcinoma [7]. Meanwhile, Isikdogan et al. reported a de novo case of mastoid osteosarcoma invading into the external ear canal without past history of radiation or Paget’s disease [8]. Finally, Strunk et al. reported a paraosteal osteosarcoma attributed to a past history of trauma [4]. Similar to these cases, our patient was managed with surgical excision of the mass. Our patient was subsequently treated with doxorubicin and cisplatin.

Osteosarcoma is one of the primary skeletal malignant tumors that function to produce immature bone tissue [11]. This type of malignancy affects 6%–13% of craniofacial bones, and usually, secondary osteosarcomas occur in the skull bones [10, 12]. Although the cause of osteosarcoma is still unclear, the only environmental factor affecting osteosarcoma in humans is ionization radiation. However, it is a rare complication of radiation therapy and usually occurs after a long latent period [13]. Sensory‐neural abnormalities may occur in cases where the lesion involves the course of peripheral nerves [14].

## 4. Conclusion

Here, we present a rare case of osteosarcoma that involved the mastoid process and invaded into the middle ear. These tumors are rare and come with unique therapeutic challenges by virtue of the anatomical location, lack of standardized treatment protocols, and poor tolerability to conventional treatment options. As such, these tumors require different treatment measures compared to typical extremity osteosarcomas. There is a relative paucity of patients with head and neck osteosarcoma. This necessitates multi‐institutional collaborative studies to devise appropriate therapeutic strategies for these tumors.

## Consent

No written consent has been obtained from the patients as there are no patient identifiable data included in this case report.

## Conflicts of Interest

The authors declare no conflicts of interest.

## Funding

The authors did not receive any specific funding for this work.

## Data Availability

The data that support the findings of this study are available from the corresponding author upon reasonable request.
